# Structural Analysis of Polymer Composites Using Spectral Domain Optical Coherence Tomography

**DOI:** 10.3390/s17051155

**Published:** 2017-05-18

**Authors:** Muhammad Faizan Shirazi, Mansik Jeon, Jeehyun Kim

**Affiliations:** 1 School of Electronics Engineering, College of IT Engineering, Kyungpook National University, 80 Daehak-ro, Bukgu, Daegu 41566, Korea; faizanshirazi110@gmail.com (M.F.S.); jeehk@knu.ac.kr (J.K.); 2 Oz-tec Co. Ltd., Office 901, IT Convergence Industrial Building, 47 Gyeongdae-ro, 17-gil, Bukgu, Daegu 41566, Korea

**Keywords:** optical coherence tomography, polymer composites, optical inspection, GPU, industrial application

## Abstract

The structural analysis of nylon/graphene oxide (NY/GO) and polyetherblockamide/ trisilinolphenyl-polyhederal oligomeric silsesquioxane (PEBA/t-POSS) composites were performed using high-resolution spectral domain optical coherence tomography (SD-OCT). This optical technology revealed both cross-sectional, as well as sub-layer depth information of sample. The non-destructive real-time imaging demonstrated the nature of defects in the composites. The thickness and location of each defect point in the composites were measured using A-scan analysis on the SD-OCT images. The cross-sectional and volumetric images clearly demonstrate the effectiveness of SD-OCT for composite research, as well as the for industrial quality assurance of polymer materials.

## 1. Introduction

Polymer composites have a vast number of applications in engineering and industrial fields due to their physical characteristics, such as high performance, light weight, chemical stability, stiffness, strength, and processability [1]. Long-term durability and reliability under different physical conditions are required for structural applications. Small defects, such as micro-cracks, delamination, and empty spaces within a layer, can affect the physical stability and shorten the composite’s lifetime. Different polymer composites demonstrated their potential use in flight vehicles, automobiles, boats, pipelines, buildings, roads, bridges, etc. Composite materials are the combination of two or more materials such that their properties are different from their constituents [2]. Since a composite material contains different materials that are responsible for their improved characteristics, the investigation of different layers below the surface of a composite has vital importance to ensure the desired properties of the composite.

By enhancing the properties of composite materials, they can also be used as selective permeable membranes for separation/purification purposes in the petroleum refining industry [3,4]. Additionally, composite materials show significant improvement in flame retardation and decomposition temperatures [5]. These properties are due to the formation of a transient protective barrier as a result of nano-sized fillers. Fundamentally, the temporary shielding to the composite is provided by the filler material that generates deposits of stable char [6,7]. The thermal stability of the nylon can be enhanced by polymer matrix of nylon-6/graphite oxide and nylon-6/graphite as demonstrated by the decomposition kinetics [8]. Similarly, the polyetherblockamide (PEBA) have applications in a wide variety of products, such as virus-proof surgical equipment, food packaging materials, antistatic sheets or belts, films for textile lamination used in sportswears and gloves, etc [9]. For different applications, the nano-sized fillers were incorporated into PEBA in order to meet certain required properties [10]. The organically-modified clay and trisilinolphenyl-polyhedral oligomeric silsesquioxane (tsp-POSS) were demonstrated in literature to enhance the properties of composites in gas separating and filtering membranes [11].

Microscopic techniques, such as scanning electron microscopy (SEM), transmission electron microscopy (TEM), scanning tunneling microscopy (STM), and atomic force microscopy (AFM), have been utilized to analyze the structural changes of composites at the micrometer and nanometer scales [12]. The principles of these techniques are different, but the goal is to produce highly-magnified surface image of the sample. However, their sample preparation, small probe areas, high cost, and the use of destructive mechanisms to extract in-depth information from them all create difficulties as the quality and extent of information also depend on the large extent of user expertise and right sample preparation. Small-angle X-ray scattering (SAXS) and wide-angle X-ray scattering (WAXS) can provide information at the atomic level [13]. However, their expensive setups make these methods less feasible for instant analysis. A couple of non-destructive methods, ultrasonic scan and X-ray computed tomography, have also been used [14,15]. Ultrasonic scans have the limitations of low resolution and the necessity of water usage as a coupling agent, while X-ray tomography is expensive for inline inspections. These techniques provide surface images at the atomic level. However, the scanning time, material preparation, extremely small probing area, and cost are all factors that limit the real-time wide-area inspection of polymer composites. In contrast, bio-photonic imaging technology can provide non-destructive sub-surface images of composite materials [16].

Optical coherence tomography (OCT) is a non-contact, non-invasive, non-destructive, and high-resolution imaging modality used for cross-sectional imaging of biological system [17]. The working principle of OCT is based on those of the Michelson interferometer, where interference is observed when light rays from the sample and reference arms are superimposed over each other. OCT is divided into two main categories; one is the time-domain (TD-OCT), while other is the Fourier domain (FD-OCT). In the time domain configuration, the reference arm oscillates with respect to time in axial direction to get coherence-gated signal from the sample and at detection end, either a single photodetector (a point scan) or an array of photodetectors (area camera) is used [18,19]. In FD-OCT, both arms are stationary compared to time domain, and the frequency encoded signal is obtained with high speed and sensitivity. The signal is decoded by a spectrometer, i.e., SD-OCT [20] or the source is a swept source, i.e., SS-OCT [21,22]. OCT employs low coherence interferometry with a broadband light source to detect the depth information of samples nondestructively. OCT is well known for its applications in biological imaging, such as in ophthalmology [20], otology [23], dermatology [24], dentistry [25], and brain studies [26]. With the advancement its application have been extended to nondestructive testing of polymer materials in industry [27,28,29,30,31], agriculture (where it is used for the detection of the quality of seeds, food, and plants) [32,33,34,35,36], and industrial products, such as light emitting diodes, liquid crystal displays, optical thin films, and touch-screen panels [37,38,39]. Furthermore, different techniques have been incorporated to improve the resolution, sensitivity, penetration depth, and speed of the OCT system [40,41,42]. This emerging photonic imaging technology is a potential alternative for instant analysis of composite materials.

In this study, nylon/graphene oxide (NY/GO) and polyetherblockamide/ trisilinolphenyl-polyhederal oligomeric silsesquioxane (PEBA/t-POSS) composites were investigated for early diagnosis of defects, such as empty cavities, lamination, and cracks, by using a high-resolution spectral-domain OCT (SD-OCT) technique. Since both composites have wide applications in different industrial fields, therefore OCT can be used as an alternative structural analysis tool for quality assurance of composite materials. Therefore, these particular composites characterization using SD-OCT is the novel approach to have the internal structure information of composites in real-time. In the laboratory, SD-OCT yielded fast and easy real-time structural information of the composites, and it can be used for inline inspection of composite materials in industry.

## 2. Materials and Methods

### 2.1. Materials

The materials used are nylon 6 (Kolon, Gwacheon, Korea), graphene oxide, polyetherblockamide (PEBA, Arkema, Birdsboro, PA, USA), trisilinolphenyl polyhedral oliomeric silsesquioxane (POSS, Hybrid plastics, Hattiesburg, MS, USA). All materials were dried in a vacuum at 120 °C for 6 h before use.

### 2.2. Synthesis of Composites

Two different types of NY/GO composite with graphene oxide (GO) contents of 5 wt % (I) and 1 wt % (II) were prepared by in situ polymerization of caprolactam in the presence of both GO and an initiator aminocaproic acid. Similarly, two types of PEBA/t-POSS composite with t-POSS contents of 5 wt % (III) and 3 wt % (IV) were prepared by melt mixing. Composites (I)–(IV) were then dried in a vacuum at 100 °C for 6 h, made into films using a hot press for 3 min set at 250 °C for composites I and II and 200 °C for composites III and IV, and finally quenched with dry ice. The thicknesses of the films were all approximately 0.50 mm.

### 2.3. Hardware and Software Setup

**Figure 1:**
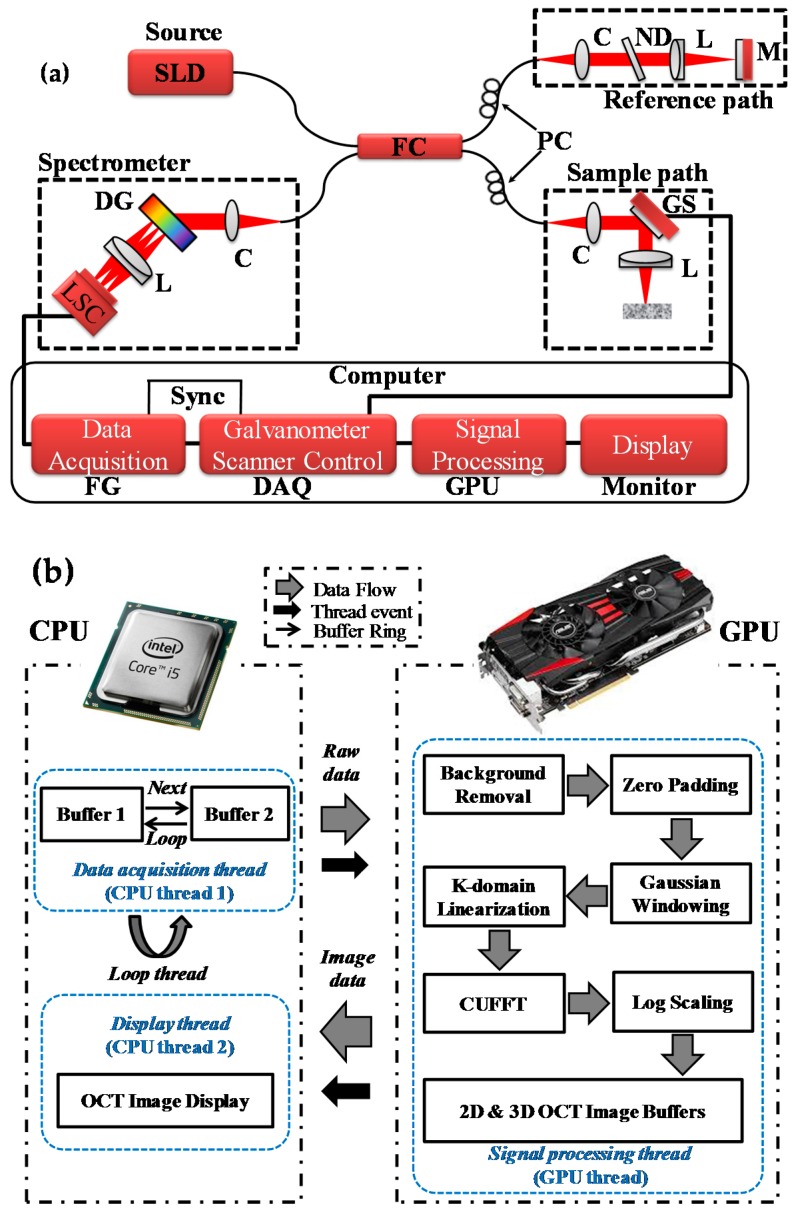
(**a**) Experimental setup, and (**b**) GPU-based signal processing algorithm for the SD-OCT. In (**a**) C: collimator, CPU: central processing unit DAQ: data acquisition board, DG: diffraction grating, FC: fiber coupler, FG: frame grabber, GPU: graphical processing unit, GS: galvanometer scanner, L: achromatic lens, LSC: line scan camera, M: mirror, ND: neutral density filter, PC: polarization controllers, SLD: super-luminescent diode.

Figure 1a shows the schematic diagram of the optical setup for the spectral domain optical coherence tomography (SD-OCT) considered in this study [33]. In brief, a broadband light source from Superlum (T-850-HP-I, Superlum, Cork, Ireland) with a center wavelength of 850 nm, a full width at half maximum (FWHM) of 165 nm, and a theoretical axial resolution of approximately 1.9 μm was utilized. The light from the source was divided equally into sample and reference paths using a 50:50 fiber coupler. The sample was scanned in the *x*- and *y*-directions using a 2D galvanometer scanner, and the reference arm’s power was controlled by a neutral density filter. The light from the fiber coupler was focused on the sample under investigation and reference mirror with identical lenses. The back-scattered/reflected light from the sample and reference paths followed the same return paths and interfered at the optical fiber coupler. This interference signal was detected using a spectrometer. The spectrometer consisted of a collimator, a diffraction grating (HD 1800 l/mm, Wasatch Photonics, Logan, UT, USA), a focusing lens, and a line scan camera (spL4096-140km, Basler, Ahrensburg, Germany). The interference light struck the diffraction grating at an optimal angle of 46°. In the first mode, light diffracted horizontally was made incident at the camera through the focusing lens. The raw signal acquired from the camera was transferred to a computer through a frame grabber (PCIe-1433, National Instruments, Austin, TX, USA). A trigger was generated with a data acquisition board (PCIe-6321, National Instruments, Austin, TX, USA) to synchronize data acquisition from the camera and the scanning frequency of the galvano-scanner.

The software algorithm used for SD-OCT signal processing is shown in Figure 1b. The data acquisition, data processing, and cross-sectional image displaying was done with LabVIEW 2013. A computer unified device architecture (CUDA) with a GTX480 NVIDIA graphics-processing unit (GPU) was utilized for high-speed data processing [39,43,44]. By incorporating GPU-based signal processing, there is approximately six times increase in the B-scan (cross-sectional image) frame rate from 10 frames/s to 60 frames/s with the image size of 2048 × 500 pixels. This high frame rate is effective for large area scanning or multiple samples scanning in lesser time for real-time measurements. GPU-based real-time signal processing enhanced the cross-sectional image acquisition speed, which resulted to acquire volumetric data in reduce time as compared to CPU based signal processing. In brief, the programming architecture of the SD-OCT system included data flow between the CPU and the GPU, event threads, and data processing. First, the data-acquisition thread stored the incoming raw two-dimensional signals into a buffer (Buffer 1) allocated in the random access memory of the CPU. After that, data were copied into another buffer (Buffer 2). This data was transferred from the buffer to the GPU memory continuously. The second buffer was employed to avoid any temporal delay in data acquisition events. The signal processing thread copied the frame data stored in Buffer 2 of the CPU memory into the GPU memory through the PCI express 2.0 × 16 bus interface. The signal processing for the OCT was divided among 480 CUDA sub-processors. The GPU-based data processing included background noise removal, zero padding, Gaussian windowing, k-domain linearization, fast Fourier transform (FFT), and log scaling. Background noise was removed by subtracting the acquired signal from the reference arm signal. Full-range k-domain linearization was used to remove the non-linearity in the raw signal [41]. A wavelength filter is utilized with a translational slit to give the selected wavelength for wavenumber linearization. The filtered spectrum with a narrow linewidth (~0.5 nm) is detected simultaneously at the spectrometer and optical spectrum analyzer (OSA, AQ6370B, Yokogawa Electric Corporation, Tokyo, Japan). The slit translates step-by-step throughout the spectrum and, at each step, the pixel position from spectrometer and respective wavelength from OSA is stored to generate a lookup table. A polynomial fitting is applied to the linearized wavenumber corresponding to all camera pixels. Finally, by utilizing this lookup table, the raw signal is linearized to remove non-linearity and, as a result, the image quality is improved.

After processing, this data was sent back to the display thread, which displayed the reconstructed OCT B-scan image in real-time. This process was sustained in order to increase the data acquisition speed with a high B-scan frame rate. The B-scan OCT images were composed of 500 × 2048 pixels, along lateral and axial directions, respectively, with a frame rate of 60 Hz. The lateral scanning range was approximately 1.5 mm × 1.5 mm by using a 2D galvanometer scanner. The volumetric image of the sample with dimension of 500 × 500 × 2048 pixels corresponding to 1.5 mm × 1.5 mm × 2 mm can be acquired in approximately 8.3 seconds, which was much faster than the CPU-based signal processing algorithm for real-time measurements. The incident power on the sample was approximately 2.5 mW. The measured lateral and axial resolutions of the system were 12 µm and 4 µm, respectively. The lateral resolution calculated by utilizing an Edmund optics lateral resolution target, while axial resolution is calculated using a point spread function in the axial direction.

## 3. Results and Discussion

**Figure 2:**
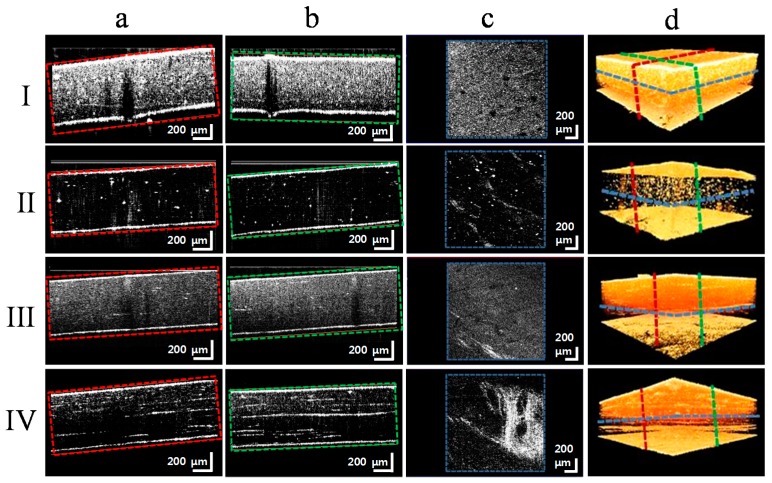
SD-OCT scan images for composites (I)–(IV) of (**a**) and (**b**) cross-sections with defects identified in lateral directions, (**c**) enface images with defects indicated, and (**d**) three-dimensional views with cross-sectional and enface image locations.

Figure 2a–d show the cross-sectional, enface, and volumetric images of composites (I)-(IV). The volumetric images were constructed from 500 B-scans. The position of the images in Figure 2a–c are indicated in Figure 2d with red, green, and blue dashed lines, respectively. Images in Figure 2a,b are respective cross-sectional images in lateral directions while Figure 2c is the enface image at certain depth in the respective composite. Since the composites were slightly tilted during imaging to avoid high back reflection as shown in Figure 2a,b. Therefore, enface images are extracted by aligning the composites in the x-y plane such that they represent the structure at a certain depth across the entire polymer below the composite surface. A defect inside the composite can be clearly observed in Figure 2a,b, while in the enface image Figure 2c, different defects can be observed at several positions. The change in the refractive index between the two components makes the defects in the SD-OCT images easily distinguishable.

The structural complexity of a polymer matrix composite requires a non-destructive inspection method to evaluate the properties of the composite. Scanning electron microscopes and transmission electron microscopes give detailed information of composites at the sub-cellular level with nanometer resolution. OCT can give real-time structure information so that the cause of a defect can be found in order to apply corrective measures in composite manufacturing. Four different composite samples were examined to obtain their cross-sectional information using a spectral domain optical coherence tomography system. The advantage of SD-OCT is that it makes real-time imaging of composite samples with high lateral and axial resolutions possible. In order to evaluate the OCT images in the axial and lateral directions, three-dimensional scanning was done on an area of interest in the sample. The cross-sectional images were rendered together for volumetric reconstruction. The enface images were extracted from the volumetric visualization at various depths so that visual information about the defects could be collected.

**Figure 3:**
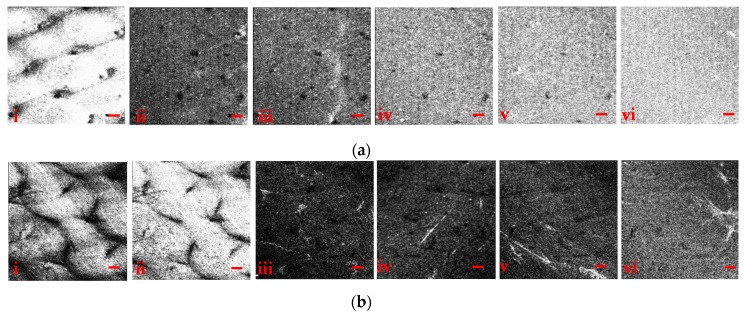
Cross-sectional enface SD-OCT images of composites (**a**) I and (**b**) III observed at progressive depth intervals of 50 μm. The scale bars are 200 µm.

Figure 3a,b show the enface depth images of composite samples I and III, respectively. The enface images were obtained at intervals of 50 μm starting from the bottom surface of each composite (Figure 3a-i and 3b-i). The defects appear as black holes at different locations in the composite samples. Moving inside the composite samples, the defects appear to penetrate in the whole composite. As can be observed from Figure 3a(ii–v), the sizes of the dark black spots are decreasing, and in (vi) these spots have disappeared. Similarly, the structural changes in surface profile and enface depth information of composite III is shown in Figure 3b. The images in Figure 3b(i–vi) are obtained at different depths, with the spots demonstrating a similar trend of decreasing in size as the one shown in Figure 3a. Some locations in the enface images show high intensities. This is due to the changes in the refractive index between the two materials where they are not mixed uniformly. The presence of lamination, micro-cracks, and micro-cavities due to improper blending and mixing of materials degrade the quality of composites.

**Figure 4:**
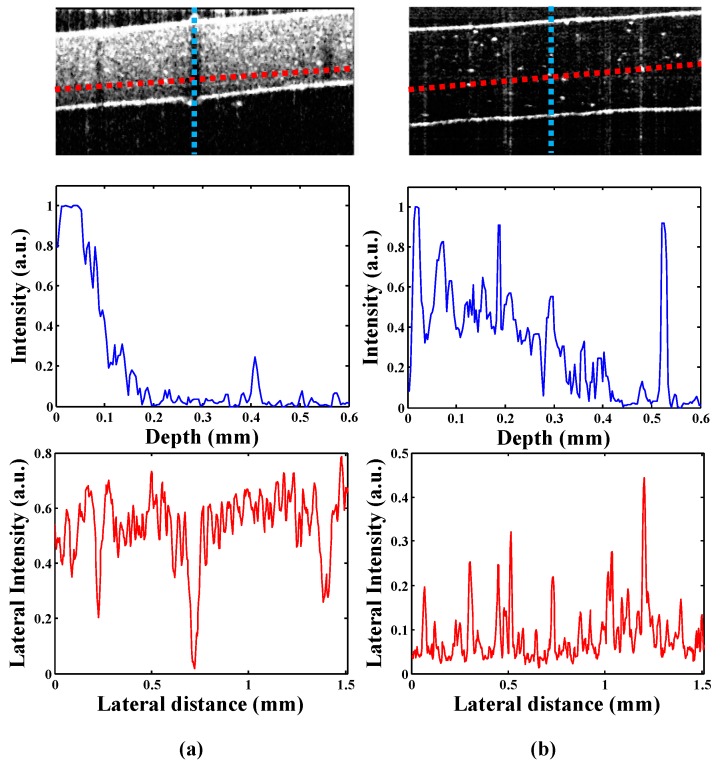

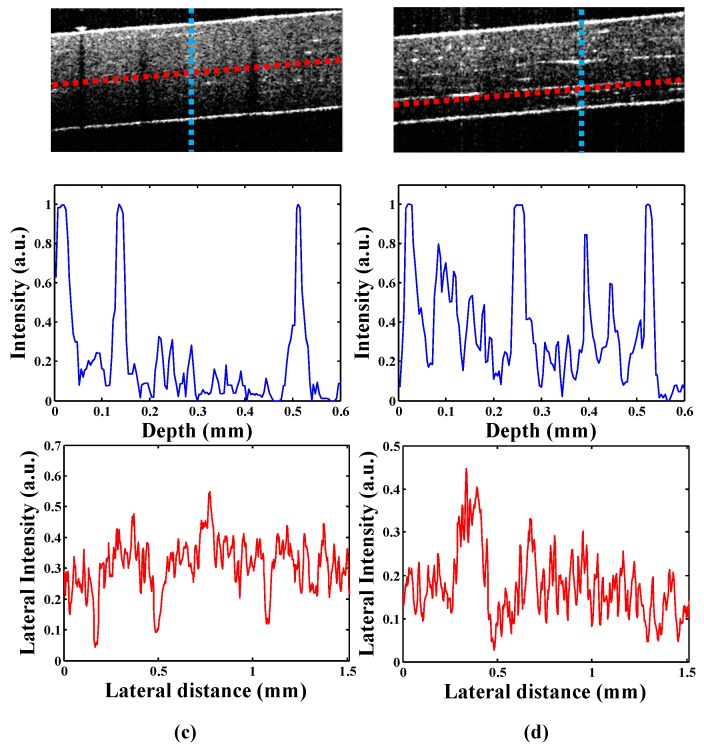
A-scan analysis of the composites in axial and lateral directions. (**a**–**d**) show the cross-sectional images of the four respective composites along with their intensity profiles in both axial and lateral directions. The blue color shows the intensity profile in axial direction, while the red color shows the intensity profile in the lateral direction.

Figure 4 shows the A-scan (amplitude-scan) analysis of four different composites in both axial and lateral directions. The distribution of material in the cross-sectional directions and the defect identification are evaluated in detail. The blue (vertical) and red (horizontal) dotted lines in the respective axial and lateral directions in the B-scan image represent region taken for A-scan analysis. Similarly, the graphs plotted in blue (top) represent the axial direction A-scans, and the graphs plotted in red (bottom) represent the lateral direction A-scans. Spatial filtering is applied on the cross-sectional images in order to remove the noise in the lateral and axial directions. The median filter with a size of 3 × 3 pixels is applied to the images to remove spiky noise in the data. For further clarity, 25 A-scans in the lateral direction were averaged to smooth out rapid fluctuations. All lateral A-scans were extracted in parallel direction to the composite surface as shown with red dotted lines. Clear correlations between the 2-D images and the calculated A-scans were revealed in both directions. In Figure 4a, the defective regions are identified in both the axial and lateral directions. The axial A-scan shows the intensity information within composite I in the defective region. The first peak in the profile indicates the first boundary of composite, which tends to decrease with no peak at the other end. In the lateral A-scan, intensity reductions at three different valleys indicated an internal defect in the lateral direction. The location of the defect showed a low intensity, which indicates the presence of improper component mixing in that region. In a polymer composite, proper mixing and uniform concentration of all components ensure the reliability, stiffness, flexibility, and long life of the final product. Figure 4b shows similar properties in composite II, since the concentration of graphene is much lower. Only a few bright spots are visible inside the composites as the result of changes in the refractive index. By employing the material distribution based on the cross-sectional image, the proportion of particles can be estimated. The axial A-scan shows an intensity profile with two peaks, indicating the top and bottom boundaries of the composite. Lateral scans are averaged to smooth down the signal and indicate the variation in brightness of signals for the defect in the center layer of the composite.

Similarly, Figure 4c,d show the cross-sectional information profiles of composites III and IV along with A-scans in lateral and axial directions respectively. As in the lateral A-scan in 4a, the lateral A-scan in 4c has a dominant intensity drop-off at three different locations. In Figure 4d, the axial A-scan has multiple peaks, which indicate that the different layers are stacked inside the composite. Intensity variations in the lateral A-scan indicate the non-uniformity of the composite material, since the mechanical properties of POSS-polymer composites are the function of the amount of POSS present in the composites. Therefore, depending on the amount of POSS present in the composites, the nanocomposites could exhibit superior or inferior mechanical properties [45]. As demonstrated in literature, the addition of 2 wt % POSS can increase 12% compressive strength, 15% flexural strength and hardness, and an unusual increase in toughness. A further addition of POSS concentration to 5 wt %, the compressive strength and modulus is increased by 31% and 68%, respectively [46]. From OCT cross-sectional images of composite III and IV with 5 wt % and 3 wt % t-POSS, the clear distinction in the composites internal structure can be observed. Composite III shows a homogenous composition, in which the intact polymer and t-POSS particles more firmly held as compared to composite IV. The presence of non-homogeneity or layer formation in composite IV can also be an indication of lower mechanical properties as compared to composite III.

**Figure 5:**
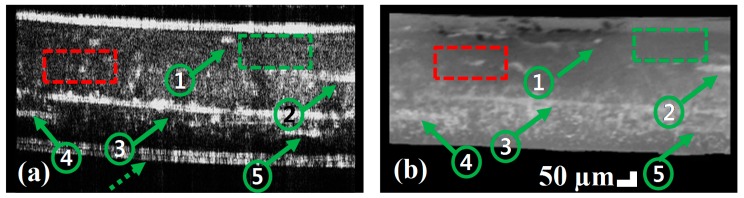
Comparison of OCT and microscopic cross-sectional images, (**a**) shows the OCT B-scan image before sectioning and (**b**) shows the microscopic image of the cross-sectional part of composite IV after sectioning at scanned position.

In order to validate the performance of SD-OCT with at the nondestructive inspection of composite material, composite IV was scanned to obtain the cross-sectional image at the marked position, non-destructively. Later, composite IV was sectioned along the marked position and the cross-sectional image was captured using a Dino-Lite digital microscope (model no. AM4515ZT4, Taipei, Taiwan). Figure 5a,b show the B-scan image from SD-OCT and microscopic image, respectively. The correlation between two images can be approximately confirmed with dominant particles and delamination, which can be seen in both images as marked by arrows 1–5. The red color dotted box indicates the non-uniformity while green color dotted box indicates the homogeneity of composite materials in both figures. Since the composite was placed on a glass coverslip for OCT imaging, the dashed arrow indicates the coverslip top layer in OCT image. The layer information in the composite can be seen in both images. The variation in the position of the layer in the OCT image is owing to the change in the refractive index of the composite material. The black color at the top layer in Figure 5b was due to the marking on the composite surface.

In future work, this study can be broadened to study the characteristics of composite in laboratory at different melting temperature. Similarly, the effect of stress can be studied using the proposed optical imaging technique. Additionally, further qualitative and quantitative inspections of composite materials can be employed using an automatic defect detection algorithm. The real-time monitoring of composites in a production line by incorporating large-scan parallel OCT systems for quality assurance can be implemented for large area scanning. Further high-resolution analysis of polymer composites can be achieved by utilizing optical coherence microscopy.

## 4. Conclusions

This study demonstrates the application of OCT as a non-destructive imaging technology in composite material investigation. The results demonstrate the quantitative and qualitative defects, such as thickness, microstructure, and physical defects, in the micrometer range of four different composites. The cross-sectional and enface information of the polymer composites indicate the potential application of OCT in the materials industry for product quality assurance. Delamination, micro-cracks, and empty spaces, which are not visible to the human eye, are detected in cross-sectional, as well as enface, images using the proposed system. In contrast to scanning electron microscopes, transmission electron microscopes, atomic force microscopes, and other kinds of technology, OCT can reveal real-time information of composite materials. By employing OCT for quality and material characteristic checks, quality assurance of the polymer-composite-based goods can be improved. Therefore, at the consumers end, reliable quality products can be obtained with satisfaction.
